# Standardization and validation of real time PCR assays for the diagnosis of histoplasmosis using three molecular targets in an animal model

**DOI:** 10.1371/journal.pone.0190311

**Published:** 2017-12-29

**Authors:** Luisa F. López, César O. Muñoz, Diego H. Cáceres, Ángela M. Tobón, Vladimir Loparev, Oliver Clay, Tom Chiller, Anastasia Litvintseva, Lalitha Gade, Ángel González, Beatriz L. Gómez

**Affiliations:** 1 Medical and Experimental Mycology Group, Corporación para Investigaciones Biológicas (CIB), Medellín, Colombia; 2 Mycotic Diseases Branch, Centers for Disease Control and Prevention, Atlanta, GA, United States of America; 3 Biotechnology Core Facility Branch, Centers for Disease Control and Prevention, Atlanta, GA, United States of America; 4 School of Medicine and Health Sciences, Universidad del Rosario, Bogotá, Colombia; 5 Cell and Molecular Biology Group, Corporación para Investigaciones Biológicas (CIB), Medellín, Colombia; 6 Basic and Applied Microbiology Research Group (MICROBA), School of Microbiology, Universidad de Antioquia, Medellín, Colombia; Oklahoma State University, UNITED STATES

## Abstract

Histoplasmosis is considered one of the most important endemic and systemic mycoses worldwide. Until now few molecular techniques have been developed for its diagnosis. The aim of this study was to develop and evaluate three real time PCR (qPCR) protocols for different protein-coding genes (100-kDa, H and M antigens) using an animal model. Fresh and formalin-fixed and paraffin-embedded (FFPE) lung tissues from BALB/c mice inoculated i.n. with 2.5x10^6^ *Histoplasma capsulatum* yeast or PBS were obtained at 1, 2, 3, 4, 8, 12 and 16 weeks post-infection. A collection of DNA from cultures representing different clades of *H*. *capsulatum* (30 strains) and other medically relevant pathogens (36 strains of related fungi and *Mycobacterium tuberculosis*) were used to analyze sensitivity and specificity. Analytical sensitivity and specificity were 100% when DNAs from the different strains were tested. The highest fungal burden occurred at first week post-infection and complete fungal clearance was observed after the third week; similar results were obtained when the presence of *H*. *capsulatum* yeast cells was demonstrated in histopathological analysis. In the first week post-infection, all fresh and FFPE lung tissues from *H*. *capsulatum*-infected animals were positive for the qPCR protocols tested except for the M antigen protocol, which gave variable results when fresh lung tissue samples were analyzed. In the second week, all qPCR protocols showed variable results for both fresh and FFPE tissues. Samples from the infected mice at the remaining times post-infection and uninfected mice (controls) were negative for all protocols. Good agreement was observed between CFUs, histopathological analysis and qPCR results for the 100-kDa and H antigen protocols. We successfully standardized and validated three qPCR assays for detecting *H*. *capsulatum* DNA in fresh and FFPE tissues, and conclude that the 100-kDa and H antigen molecular assays are promising tests for diagnosing this mycosis.

## Introduction

Histoplasmosis is a disease caused by the dimorphic fungus *Histoplasma capsulatum*, which is considered one of the most important endemic mycoses and has caused outbreaks worldwide [1]. This disease is most frequently diagnosed in the Americas. The infection is acquired via the respiratory route, particularly when soils enriched with contaminated bird or bat excreta are disturbed, giving rise to infectious aerosols [2]. Clinical manifestations are variable and are especially severe in immunosuppressed patients, especially those living with HIV/AIDS, in whom histoplasmosis presents with significant morbidity and mortality [3], especially in those countries with limited access to rapid diagnostics or antiretroviral therapies, with up to 48% mortality reported [4,5].

Conventional laboratory methods used for diagnosis of histoplasmosis, such as blood cultures and histopathological analysis with visualization of the small yeast in fresh or formalin-fixed paraffin embedded (FFPE) tissues, have variable sensitivity (~50%) [2,3] and cultures may take up to 6 weeks to grow, delaying considerably the diagnosis [6,7]. Immunological tests that detect antibodies are less sensitive when used in immunocompromised persons, who often cannot mount a good humoral immune response [8]; a sensitivity of immunological tests has been reported ranging between 38% and 70% [6]. Detection of circulating *Histoplasma* antigens in urine has proven highly sensitive (95%) [5,9,10], but this test is not widely available; therefore each test has limitations [11].

A few molecular approaches have been developed by different groups and are all “in house” methods [4,12–17]. The most common target used for molecular diagnosis of histoplasmosis has been the gene coding for a specific 100-kDa protein reported as essential for the survival of *H*. *capsulatum* in human cells [18]. Many groups have reported the 100-kDa protein as a molecular target using quantitative and qualitative assays via PCR in fresh and FFPE tissues, as well as the loop-mediated isothermal amplification (LAMP) test [14,15,19–22]. The small-subunit rRNA (18S) gene of *H*. *capsulatum* has also been employed in nested-PCR and quantitative real time PCR (qPCR) assays [13,23,24]. The genes encoding the H and M antigens have also been used as molecular targets, as they both are specific proteins widely used for immunodiagnostic assays; M antigen target has been described by Guedes *et al* using a conventional PCR, and H antigen by Bracca *et al* in a nested PCR, both with specificity and sensitivity above 90% [25,26]. Currently, DNA-based diagnostic tests are not yet established as a routine diagnostic tool for histoplasmosis, universal molecular targets have not been established and some proposed tests have not been validated in human samples; for those reasons, at present no consensus molecular diagnostic test has been established and no PCR assays are commercially available.

Animal models are important in order to evaluate the performance of molecular biology based tests for identifying fungal DNA and they can also help to understand the kinetics of fungal DNA during infection. Very few murine models (two reports in the literature) have been described for validating a nested PCR assay [23,24], but none have been described for quantitative real time PCR assays.

Thus, there is an important and urgent need to develop new and rapid molecular assays for the diagnosis of histoplasmosis with high sensitivity and specificity, similar to those currently used for other pathogens such as *Aspergillus* spp [27–29]. The aim of this study was to design and evaluate quantitative real time PCR (qPCR) protocols targeting three protein-coding genes (100-kDa protein, H and M antigens) in a controlled histoplasmosis infection using an animal model.

## Materials and methods

### Strains and DNA collection

A collection of 30 DNAs extracted from cultures of *H*. *capsulatum*, representing six different clades described so far [30], and 36 DNAs extracted from medically relevant pathogens were used to evaluate the performance of the new designed qPCR assays (Tables 1 and 2). Isolates were provided by the Mycotic Diseases Branch (MDB), CDC, Atlanta, USA and the Corporación para Investigaciones Biológicas (CIB), Medellín Colombia. DNAs of different *H*. *capsulatum* clades and from medically relevant pathogens were quantified and adjusted to 1 ng/μl of concentration for the assays. In addition, mouse DNA (Promega, Madison, WI, USA) was used. Genomic DNA extraction process from the strain culture collection was performed using QIAamp® DNA Mini Kit (Qiagen; Valencia, CA, USA) as described by the manufacturer.

**Table 1:** Collection of DNA from cultures of *H*. *capsulatum* representing different clades.

Species	Isolate name	Variety
NAm1	1001	*capsulatum*
	2212	*capsulatum*
	2763	*capsulatum*
	2771	*capsulatum*
NAm2	1000/H8	*capsulatum*
	1003/H11	*capsulatum*
	1006	*capsulatum*
	1008	*capsulatum*
	2404/H77	*capsulatum*
	2434/H84	*capsulatum*
	2436/H86	*capsulatum*
	2472/H97	*capsulatum*
	2474	*capsulatum*
	2475	*capsulatum*
LAmA	2134	*capsulatum*
	2350/H60	*capsulatum*
	2352/H62	*capsulatum*
	2353/H63	*capsulatum*
	2355/H73	*capsulatum*
	2358/H67	*capsulatum*
	2367/H76	*capsulatum*
LAmB	2349/H59	*capsulatum*
	2359/H68	*capsulatum*
	2363/H70	*capsulatum*
	2365/H75	*capsulatum*
	2368	*capsulatum*
Netherlands	4741/H176	*capsulatum*
Africa	2444/H91	*duboisii*
	5822	*duboisii*
	5823	*duboisii*

**Table 2:** Collection of DNA from cultures of medically relevant pathogens.

Strains	Strain source
*Aspergillus fumigatus*	IFI 03–0127
*Aspergillus ustus*	IFI 01–0058
*Aspergillus niger*	IFI 03–0235
*Aspergillus terreus*	IFI 02–0228
*Aspergillus versicolor*	NRRL 236
*Aspergillus flavus*	IFI 01–0074
*Fusarium moniliforme*	B6584
*Fusarium solani*	B5730
*Fusarium oxysporum*	B6936
*Rhizopus microsporus*	B4209
*Rhizopus arrhizus (oryzae)*	B4386
*Absidia corymbifera*	B6587
*Cunninghamella elegans*	NRRL 28624
*Apophysomyces elegans*	NRRL 28632
*Syncephalastrum racemosum*	B6101
*Penicillium marneffei*	B6471
*Penicillium* sp.	B5449
*Penicillium* sp.	B5454
*Scedosporium apiospermum*	B5400
*Chrysosporium keratinophilum*	B1980
*Candida albicans*	CAS 12–3800
*Candida glabrata*	CAS 99–236
*Candida parapsilosis*	CAS 10–2433
*Candida krusei*	CAS 11–3417
*Candida tropicales*	CAS 11–2787
*Candida dubliniensis*	CAS 98–174
*Candida lusitaniae*	CAS 11–3483
*Cryptococcus neoformans*	2012–820513
*Cryptococcus gatti (D)*	SA 03–0241
*Sporothrix schenckii*	B3759
*Blastomyces dermatitidis*	2010–24457
*Blastomyces dermatitidis*	B3591
*Coccidioides immitis*	2010–18016
*Coccidioides posadasii*	2011–02345
*Paracoccidioides brasiliensis*	PB 339
*Mycobacterium tuberculosis*	H57Rv

### Primer and probe design

Known sequences of the three important *H*. *capsulatum* protein-coding genes (100-kDa, H and M antigens) were retrieved from the GenBank database (accession numbers: AJ005963.1, U20346.1 and AF026268.2) and sequences for each of the three genes were aligned using the Clustal X program. The alignments were enriched by additional in-house sequencing of more isolates from each of the known *H*. *capsulatum* clades [30], and then scanned for regions of high intraspecies sequence conservation that could be used for designing primers and probes (S1–S3 Files).

All primers and probes were designed *de novo* and tested with Blast (Basic Local Alignment Search Tool; NCBI). No matches to other microorganisms or human DNA were observed. TIB MOLBIOL, Adelphia, NJ, USA was consulted for fine-tuning of the designs.

### Real time PCR assay

Primers and probes were standardized using different concentrations (0.1μM, 0.2μM and 0.3μM) and two annealing temperatures (58°C and 60°C). Optimal conditions for each protocol were chosen as follows: the qPCR was performed in a mixture (25μl) that contained 1X Maxima Probe qPCR Master Mix (2X) (Thermo Fisher Scientific, USA), 0.2μM primers for the 100-kDa and M antigen protocols, and 0.3μM primers for H antigen protocol, 0.3μM probe for all protocols and 1 ng genomic DNA. Primer and probe sequences are shown in Table 3. qPCR was performed using a Rotor-Gene 6000 thermocycler (Qiagen; Valencia, CA, USA) under the following conditions: 95°C for 10 min, followed for 50 cycles of 95°C for 15 sec, 58°C for 30 sec and 72°C for 10 sec. The amplicons generated had lengths of 90 bp, 104 bp and 173 bp for the 100-kDa, H and M antigen protocols, respectively. Positive (*H*. *capsulatum* DNA) and negative controls (sterile distilled water) were included in each qPCR assay. Additionally, conventional PCR using the actin housekeeping gene was performed to assess the presence of amplifiable murine DNA and to detect PCR inhibitors following the protocol described by Bialek *et al* [23].

**Table 3:** Sequences of the primers and probes for each molecular target.

Primers and probes	Sequence 5’-3’	Protein-coding genes
84R22 (Forward)	5`GCAGARAATTCGACCYCAAGCC 3’	100-kDa
15F23 (Reverse)	5`GTATCCCACAGCATCMYGGAGGT 3’	
45L23	5`6FAM-CCTTCTTGCAACTYCCYGCGTCT-BBQ 3’	
2F (Forward)	5`CACCGAGATAAAGGTGTCGA 3’	H Antigen
2R (Reverse)	5`GGATCAGGGCTGAAACCTTC 3’	
2P	5`6FAM-CTGCCCAACGGTCCAACCACA-BBQ 3’	
F1 (Forward)	5`CGCCRACGGGCTTTCCAT 3’	M Antigen
R1 (Reverse)	5`CATCGCTTGATCCCCAACG 3’	
TM	5`6FAM-ACCYSTTGGGTATTGCGTTGAGG-BBQ 3’	

### Determining analytical sensitivity

The PCR products were cloned in TOPO^®^ TA Cloning Kit (Thermo Fisher Scientific, USA). All PCR products were inserted into pCR™4-TOPO^®^ vector and transformed into TOP10 chemically competent *Escherichia coli* cells. Plasmids were extracted using PureLink® Quick Plasmid Miniprep (Thermo Fisher Scientific, USA).

Analytical sensitivity was obtained by serial dilution of *H*. *capsulatum* plasmid DNA (10ng until 1fg of DNA/μl). A standard curve and the crossing points (threshold cycle) were obtained in order to calculate copy number for each qPCR protocol.

### Mouse model of pulmonary histoplasmosis

#### *H*. *capsulatum* strain

*Histoplasma capsulatum* yeasts (CIB1980 strain) were cultured in 200ml Brain Heart Infusion (BHI) broth medium (BDBBL, Franklin Lakes, NJ, USA) supplemented with 10% glucose (Sigma-Aldrich, Saint Louis, MO, USA), 0.1% L-cystine (Sigma-Aldrich, Bogotá, Colombia) and 100 U/ml Penicillin-100 μg/ml Streptomycin (GIBCO Invitrogen Corporation, Carlsbad, CA, USA) at 36°C and 150 rpm for 48 hours. Yeasts were centrifuged at 25°C, 3000 rpm during 5 min, the pellet was resuspended in 100μl of 1X phosphate-buffered saline (PBS) (GIBCO Invitrogen Corporation, Carlsbad, CA, USA) and the yeasts were counted using a haemocytometer and adjusted to 2.5x10^6^ cells contained in 60μl in order to infect the mice.

#### Recovery of fungal virulence of *H*. *capsulatum* strain

BALB/c mice (n = 3) were intravenously (i.v.) inoculated through the tail vein with 5x10^6^ viable yeast cells of *H*. *capsulatum* isolate 1980. After 4 weeks, mice were euthanized and affected organs (spleen, liver and lungs) extracted. The organs were macerated and cultured at 37°C in BBL Brain Heart Infusion (BHI) supplemented agar for two weeks. The *H*. *capsulatum* virulence-recovered yeast phase was subcultured at least twice weekly and then used for the intranasal (i.n.) inoculation of animals.

#### Animals and *H*. *capsulatum* infection

BALB/c mice were provided from the breeding colony maintained at Corporación para Investigaciones Biológicas (CIB, Medellín, Colombia). Mice were housed in the animal facility at the CIB in a caging system (RAIR HD Super Mouse 750^TM^ Racks system, Lab Products, Inc. Seaford, DE, USA) equipped with high efficiency particulate air (HEPA) filters with controlled room temperature at 20–24°C; and under sterile conditions and provided with sterilized food and water *ad libitum*. Seven-week-old male mice were used in this study. Two experimental groups of mice were gathered, which consisted of infected or non-infected control mice (n = 140). Each group consisted of 70 animals, and from these, 10 mice were used for each post-infection period evaluated (1, 2, 3, 4, 8, 12 and 16 weeks). Infected mice were challenged intranasally with 60μl of 2.5x10^6^ *H*. *capsulatum* yeast cells, and mice were previously anesthetized via intramuscular injection of 50μl Ketamine-Xylazine solution at 80mg/kg Ketamine (Laboratorios Biosano, Santiago, Chile) and 8mg/kg Xylazine (Bayer S.A., Bogotá, Colombia). Mice used as controls were inoculated with 60μl of PBS 1X under the same conditions. Cages were checked once daily for dead or moribund mice and no animals were found dead prior to sacrifice at the periods evaluated; every effort was made to minimize suffering of animals. Mice were euthanized at the different evaluation periods described above, chosen as the final point, using anesthesia followed by cervical dislocation. Lungs were removed for quantification of fungal burden by colony-forming units (CFU) and DNA extraction (5 mice per period), and histopathological analysis (5 mice per period). The number of animals used in this study was rationally chosen to obtain statistical significance in accordance with other studies described in the literature [31–33].

#### Ethics statement

This study was performed according to recommendations of European Union, Canadian Council on Animal Care, and Colombian regulations (Law 84/1989, Resolution No. 8430/1993). The protocol was approved by the Ethics in Research Committee of the CIB (Comité de ética, Act No. 098).

#### Colony forming units (CFU) determination

Lungs were removed, homogenized and processed following the protocol described by Pino-Tamayo *et al* with some modifications [31]. Briefly, lungs were weighed and homogenized in 2ml sterile PBS/ 1% Penicillin–Streptomycin solution [(GIBCO Invitrogen Corporation, Carlsbad, CA, USA) to a final concentration of 100 U/ml Penicillin– 100 μg/ml Streptomycin] using a gentleMACS Dissociator (Miltenyi Biotec, Teterow, Germany). Homogeneous suspensions were diluted (10^1^ to 10^4^) and 0.5ml of each dilution was plated on duplicate petri plates with Brain Heart Infusion (BHI) agar medium (BDBBL, Franklin Lakes, NJ, USA) supplemented with 10% glucose, 0.1% L-cystine, 100 U/ml Penicillin-100 μg/ml Streptomycin and 5% sheep erythrocytes (MDM Científica, Medellín, Colombia). Incubation was done at 36°C, 5% CO_2_ for 15 days. The CFU count was calculated as previously described [31]. The remaining homogenized tissues were stored at -20°C until DNA extraction.

#### Histopathological analysis

Lungs of mice were recovered and processed for histopathological analysis following the protocol described by Pino-Tamayo *et al* [31] with some modifications. Briefly, lungs were perfused with 1X PBS to wash out red blood cells, followed by formalin solution to fix tissues [Formaldehyde solution, (EM Science, Gibbstown, NJ) to a final concentration of 4%, sodium dihydrogen phosphate (Merck, Darmstadt, Germany) to a final concentration of 0.15M and sodium hydroxide (Sigma-Aldrich, Saint Louis, MO, USA) to a final concentration of 0.11M]. Lungs were removed and submerged in 4% formalin for a maximum of 24 hours. Fixed tissues were embedded in paraffin (FFPE) and cut. FFPE tissue sections were stained with Grocott’s methenamine silver to identify *H*. *capsulatum* yeast cells. Tissues were photographed using a Nikon Eclipse Ci-L microscope (Nikon Instruments Inc.,Melville, USA) and a Nikon DS-Fi2 high-definition color digital camera, and analyzed using NIS Elements 4.30.02 Laboratory Image Software (Nikon Instruments Inc., Melville, USA) and ImageJ Software (National Institutes of Health, NIH, Maryland, USA).

#### DNA extraction from lung tissues

Nucleic acids were extracted from fresh lung tissues and FFPE samples using QIAamp® DNA Mini Kit (Qiagen; Valencia, CA, USA) and QIAamp® DNA FFPE Tissue Kit (Qiagen; Valencia, CA, USA) respectively, according to the manufacturer’s instructions and modifications described previously [34].

### Statistical analysis

Data analysis was performed using Graph Pad Prism software version 7 (GraphPad Software, Inc., La Jolla, CA, USA). Fungal burden was expressed as the mean ± SD number of CFU per g of tissue. In order to determine the agreement between the qPCR and either direct examination or culture (CFU), *Kappa* analysis was performed, using the statistical software STATA version 11.

## Results

### Design of primers and probes for the three *H*. *capsulatum* gene targets

Different primers and probes were initially designed for each of the three molecular targets. A total of 5 probe and 13 primer locations were considered for the genes coding for the 100-kDa protein and H and M antigens (full alignments are shown in S1–S3 Files). Three qPCR protocols were selected, for the *H*. *capsulatum* protein-coding genes for 100-kDa, H and M antigen, respectively, and the best signal for each target was determined using different combinations of melting temperatures and primer and probe concentrations (Table 3). No other microorganisms or human DNA had sequence matches with primers/probes. No amplification signals were detected in the negative controls.

### Analytical sensitivity and specificity of the molecular assays

**Fig 1:**
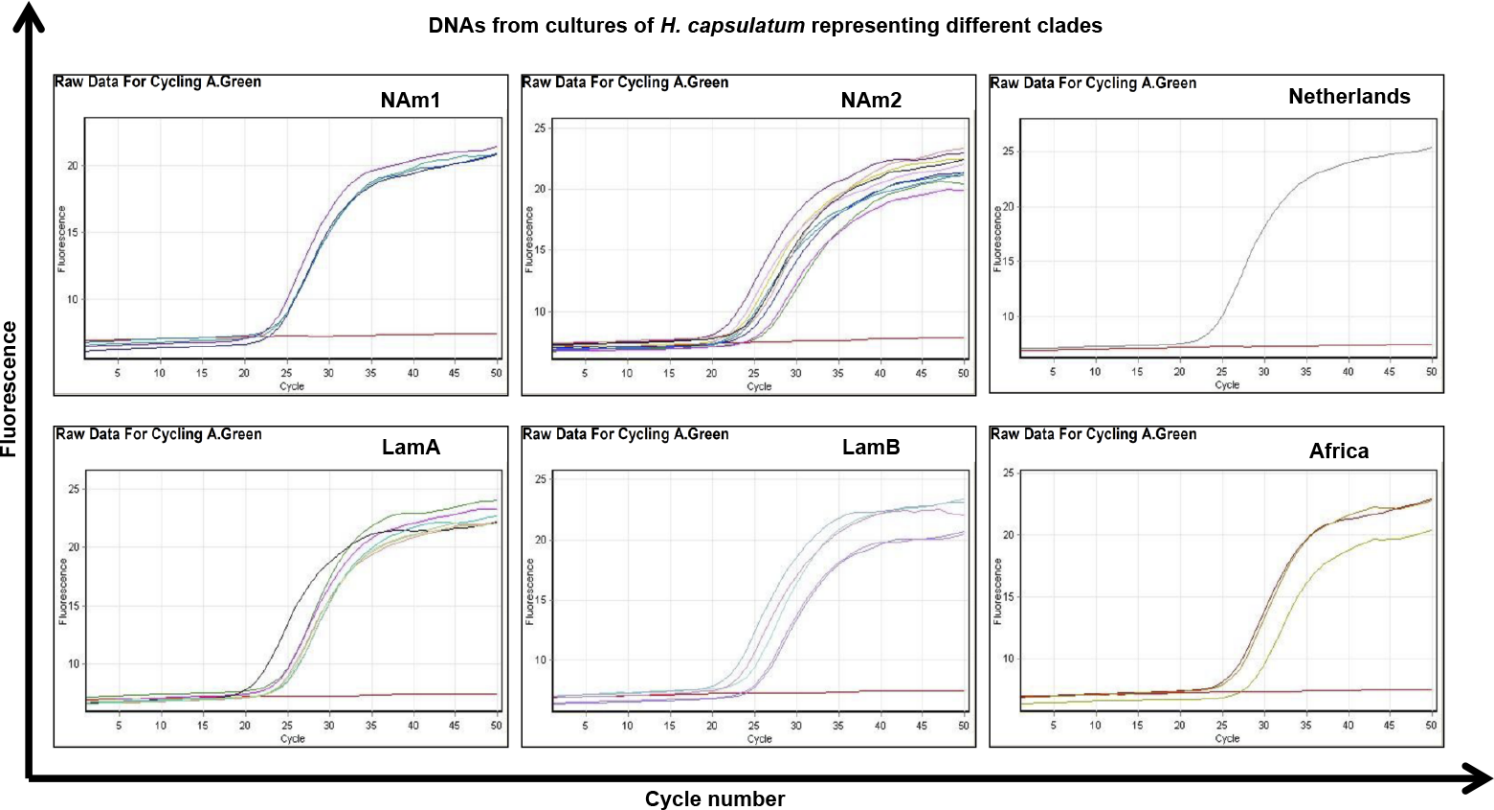
Standardization of qPCR protocols: Sensitivity of the real time PCR using 30 different *Histoplasma* DNAs representing 6 of the 8 clades described. Example of 100-kDa qPCR protocol. NAm1: n = 4, Nam2: n = 10, LamA: n = 7, LamB: n = 5, Netherlands: n = 1, Africa: n = 3.

**Fig 2:**
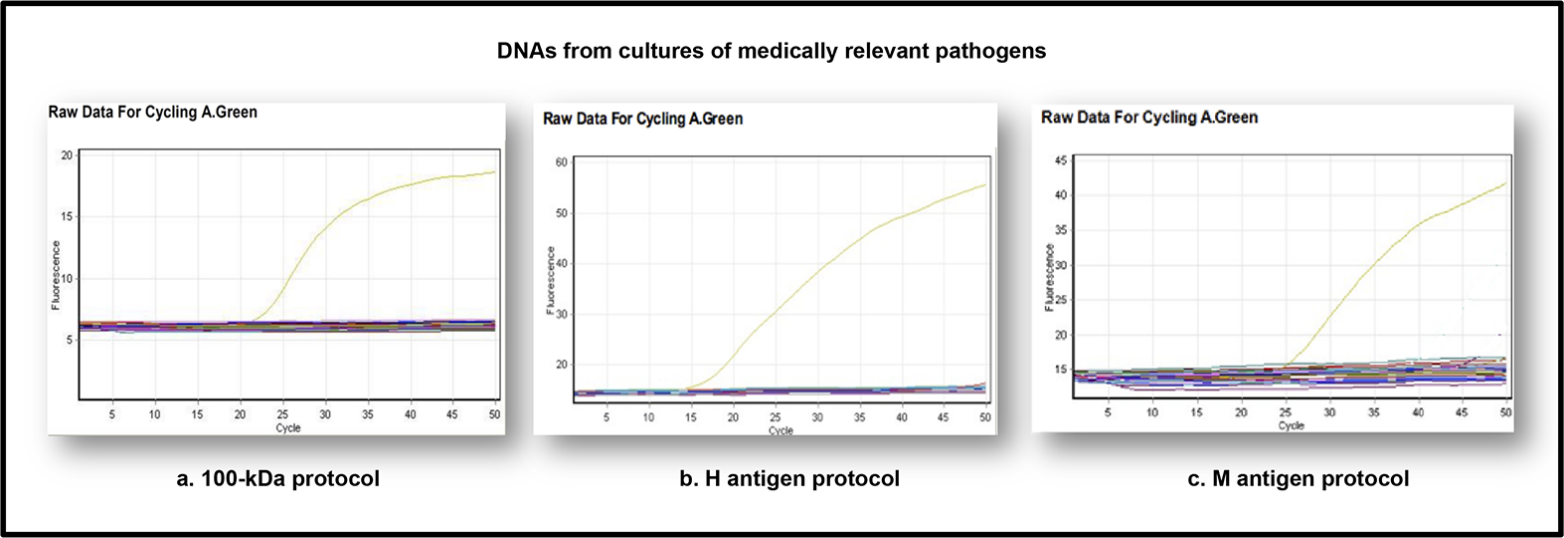
Standardization of qPCR protocols: Specificity of the real time PCR using DNAs from other important medical pathogens. Example of 100-kDa, H and M qPCR protocols, testing fungal and *Mycobacterium* DNA from 36 reference isolates. Signal is observed with *H*. *capsulatum* DNA (yellow line), the remaining samples are negative.

DNAs extracted from 66 strains were used to evaluate the performance of the real time PCR assay. From these, the 30 DNA *H*. *capsulatum* strains representing the different clades described by Kasuga *et al* [30] were all positive when tested using the three protocols (Fig 1) and C_*t*_ values are presented in Table 4. The DNA from the 36 strains from other medically relevant pathogens were all negative (Fig 2). The absence of cross-reaction with mouse DNA was also confirmed.

**Table 4:** Average of crossing point (threshold cycle) for each *H*. *capsulatum* clade for the three qPCR protocols.

*H*. *capsulatum* clades	C_*t*_ value of real time PCR protocols		
	100-kDa	H antigen	M antigen
**Nam1**	20	20	24
**Nam2**	20	22	24
**LamA**	21	23	25
**LamB**	21	23	24
**Netherlands**	21	23	24
**Africa**	24	24	27

The detection limit was 1 fg of DNA per μl of sample, which needs 2, 1 and 43 DNA copies/μl for the 100-kDa, H and M antigen protocols respectively, when the coefficient *r*^*2*^ of the linear regression between crossing-point values and different dilutions of genomic DNA was determined (*r*^*2*^ > 0.99). The analytical sensitivity assay was performed with a single round of PCR.

### Fungal burden and histopathological analysis of the lungs

**Fig 3:**
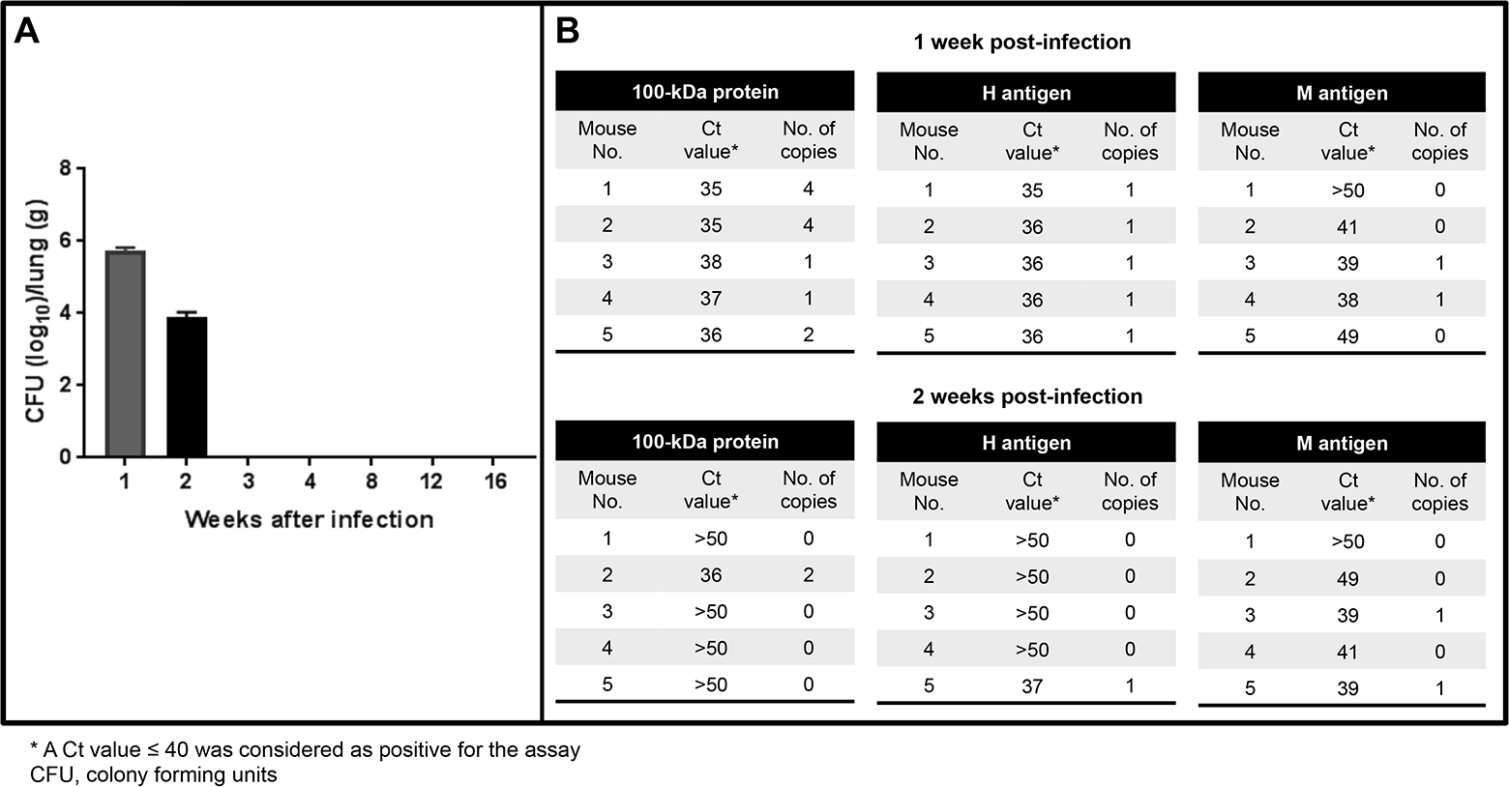
Fungal burden and detection of *Histoplasma* DNA in fresh lung tissues using the three molecular targets. A) Quantitative analysis of colony forming units (CFUs) evaluated in lungs of mice challenged i.n. with 2.5x10^6^ *H*. *capsulatum* yeast cells at different weeks post-infection. B) Results of qPCR represented with Ct values and number of copies for each protocol at the first and second weeks post-infection.

Animal infection was confirmed by analyzing the CFUs, which showed infection (fungal burden) in the lungs during the first two weeks post-infection. The highest fungal burden occurred at first week post-infection and full fungal clearance was observed from the third week (Fig 3A). Cultures of lungs were negative in the remaining periods studied. Mean fungal burden was 5.7 CFU (± 0.2 Log_10_/g of tissue) and 3.9 CFU (± 0.2 Log_10_/g of tissue) in the first and second week, respectively (Fig 3A). A statistically significant reduction in the fungal burden was observed in the lungs of infected mice when the first week post-infection was compared with the other periods evaluated (*P* < 0.00001).

**Fig 4:**
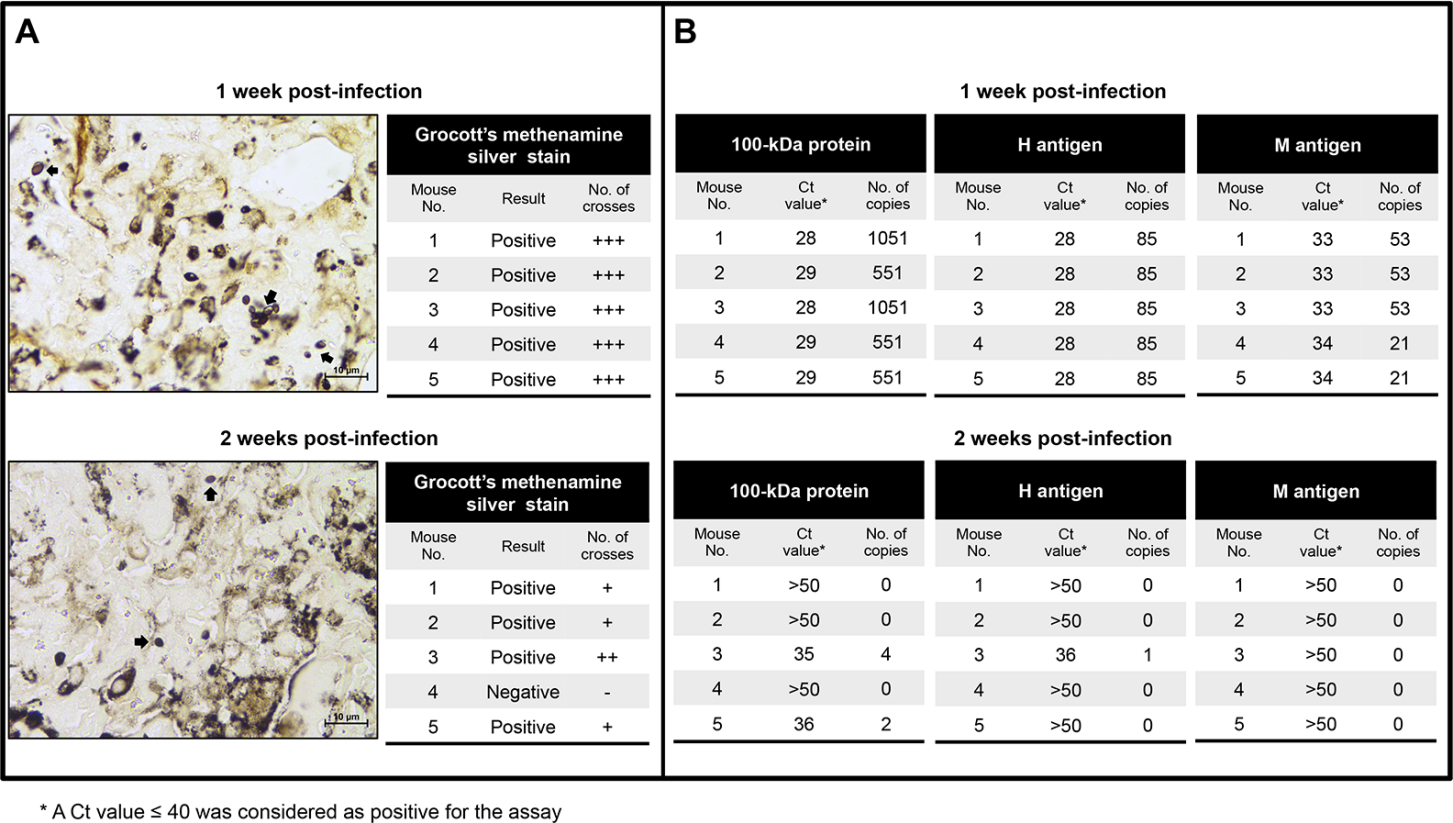
Histopathological analysis and detection of *Histoplasma* DNA in FFPE lung tissues using the three molecular targets. A) Microphotographs from lung sections stained with Grocott’s methenamine silver from mice infected with 2.5x10^6^ *H*. *capsulatum* yeast cells at the first and second weeks post-infection, with the respective semiquantification measured by crosses of *H*. *capsulatum* yeast cells. Magnification 100X. B) Results of qPCR are represented with Ct values and number of copies for each protocol at the first and second weeks post-infection.

Histopathological analysis of lung tissues from infected mice demonstrated the presence of *H*. *capsulatum* yeast cells only during the first two weeks, as was observed for the CFU. In the first week, all the FFPE tissue sections showed fungal cells in high quantity (+++) (Fig 4A), while in the second week post-infection 4 out of 5 mice showed low numbers of yeast cells (+ to ++) and one was negative (Fig 4A).

### Detection of *Histoplasma* DNA in the lungs of mice

Real time PCR was performed in order to detect and quantify *H*. *capsulatum* DNA in fresh and FFPE lung tissue samples.

Molecular assays were positive for all fresh lung tissue samples at the first week post-infection for the 100-kDa protein and H antigen protocols with a number of copies detected ranging from 1 to 4, while only two of five samples were positive for the M antigen protocol with 1 copy detected (Fig 3B). Of note, at the second week post-infection only one sample was positive for the 100-kDa protein (2 copies detected), one for the H antigen (1 copy detected) and two samples were positive for the M antigen protocol (1 copy for both samples) (Fig 3B). These real time PCR assays showed good agreement with the CFU results for the 100-kDa protein and H protein protocols (*Kappa* = 0.68 for both) and not good agreement for the M antigen protocol (*Kappa* = 0.49); it should be kept in mind that CFU results shown in Fig 3A are on a logarithmic scale.

Interestingly, at the first week post-infection all the FFPE lung tissue samples from infected mice were positive for all three protocols tested, with number of detected copies ranging from 21 to 1051 (Fig 4B), whereas in the second week post-infection only two samples were positive for the 100-kDa protein (with 2 and 4 copies detected), one for the H antigen (1 copy detected) and none for the M antigen protocol (Fig 4B). Comparison between real time PCR assays for the 100-kDa protein and histopathological analysis with FFPE samples showed a very good agreement (*Kappa* = 0.84), and similar but lower agreements were observed when the H and M antigen protocols were analyzed (*Kappa* = 0.75 and 0.65 respectively).

A sample was considered positive for the assay when a Ct value was ≤ 40. This crossing point (threshold cycle) was selected based on experience in our research groups, on the evaluation of qPCR conditions and on studies in the literature that focused on identifying other fungal pathogens in animal models [35,36]. No signal amplification was detected for fresh and FFPE lung tissue samples in the remaining periods evaluated. All samples from uninfected mice (controls) were negative for all three protocols evaluated. In order to determine the presence of PCR inhibitors, the β-actin DNA gene was amplified and was positive for all lung samples tested indicating the absence of PCR inhibitors.

## Discussion

We have developed and evaluated three real time PCR assays for the detection of *H*.*capsulatum* using different protein-coding gene targets (100-kDa, H and M antigens). To our knowledge, they are the first real time PCR assays reported for this pathogen to be evaluated using an animal model, BALB/c mice inoculated via the intranasal route with *H*. *capsulatum* yeast cells.

Protein-coding genes were selected as molecular targets on the basis of their established diagnostic importance. In the case of the 100-kDa target, the protein is essential for the survival of *H*. *capsulatum* in human cells [18] and has been used in a nested PCR molecular assay for detecting the fungus in animal and human samples for many years [15–17,19–22]. H antigen is a ß glycosidase and M antigen a catalase that have been used for detecting antibodies against *H*. *capsulatum* in body fluids in routine diagnosis for decades [7,37], however there have been few reports of their use in molecular assays and none for real time PCR assays [25,26].

We designed *de novo* for the first time three protein-coding gene primer/probe sets for detecting DNA of *H*. *capsulatum* in real time PCR and showed 100% specificity and analytical sensitivity, with a high detection limit of copies for 100-kDa, H and M antigen protocols (2, 1 and 43 DNA copies/μl respectively). The three assays were able to detect DNA extracted from *H*. *capsulatum* isolates from 6 different clades, as shown in Fig 1 for the 100-kDa assay as an example. The different clades were taken into consideration in the assay designs with the purpose of detecting *H*. *capsulatum* DNA distributed worldwide, although more studies using animal or human tissues infected with different clades will be need to verify the capacity of “universal detection”.

There have been very few reports of animal models for validating diagnostic tests, and those were typically for aspergillosis or candidiasis [27,38–40]. Animal models for histoplasmosis have also been developed, but not used for quantitative molecular assays [19,23]. In our study, fungal burden on lung tissues showed infection during the first two weeks with 5.7 CFU (Log_10_/g of tissue) and 3.9 CFU (Log_10_/g of tissue), respectively and clearance was observed from the third week (Fig 3A); clearance presumably indicates control of the infection in BALB/c mice. Our findings are in agreement with another study reported by Bialek *et al* [23], who showed fungal burden peaking on day 5 and declining thereafter, and with cultures being completely negative from day 15 onwards, in lungs of ICR mice.

In this study, results of histopathological analysis were similar to those described for CFU. *Histoplasma capsulatum* yeast cells were observed only during the first two weeks; in the second week the number of yeast cells decreased and was negative at third week, a finding that indicates a clearance of infection. Other studies have been performed using an animal model but no histopathological analysis was done [23] or the analyses performed were of other sample types such as spleen tissues [24].

The 100-kDa and H antigen molecular assays were positive for all fresh and FFPE lung tissue samples at the first week post-infection, at the second week post-infection a few samples were still positive (Figs 3B and 4B), and all samples were negative from the third week, as was observed also in the CFU and histopathological analyses. Our real time PCR results using the 100-kDa and H antigens protocols showed a good agreement with CFU and histopathological analysis results, particularly in FFPE lung tissues, where a higher number of copies were detected and where also the M antigen protocol gave excellent and reliable results. It may be asked why the M antigen assay failed to detect infection in fresh tissues at first week post-infection, and we consider as a possibility that the longer amplicon length of this assay (173bp vs 104 and 90bp) may cause difficulties when samples contain partly degraded DNA.

With respect to CFU and histopathological analyses, no previous study has compared the results with quantitative molecular assays such as real time PCR.

Our results using the qPCR assay for the detection of the 100-kDa protein and H antigen are concordant with those of Bialek *et al* [23] using the nested PCR for the small-subunit (18S) rRNA gene as a target with good correlation measured by CFU. A year later the same group [24] compared the nested PCR results with histopathological stain (Grocott) and reported the nested PCR as the most sensitive method but not significantly more sensitive than the Grocott stain. We also found that our real time PCR protocols had very good agreement with the Grocott stain.

The sensitivity of our qPCR assay using the actin housekeeping gene in order to detect the presence of amplifiable murine DNA and to detect PCR inhibitors in fresh and FFPE tissues was 100%. With this result we demonstrate that a controlled fixation of the tissues, a proper storage time and adequate temperature process are important in order to obtain DNA of high quality and amplifiable via PCR methods. Some authors have demonstrated the negative effect of formalin fixation during the process due to cross-linking and fragmentation of DNA [19,34,41,42], for that reason other authors have been working to improve the protocols [34,43].

## Conclusions

We successfully standardized and validated three qPCR assays for detecting *H*. *capsulatum* DNA in a collection of DNA from different clades and also in fresh and FFPE tissues from infected animals. Our results showed that the 100-kDa and H antigen molecular assays both have high sensitivity for detecting *Histoplasma capsulatum* DNA in tissues from infected mice. We suggest that these novel molecular assays are promising tests for diagnosing this important mycosis in clinical samples.

## Supporting information

S1 File Sequences from different *H*. *capsulatum* clades used for designing primers and probes used for 100-kDa protocol. The final choices of primer and probe sequences are shown highlighted in yellow and turquoise, respectively.
(PDF)

S2 File Sequences from different *H*. *capsulatum* clades used for designing primers and probes used for H antigen protocol. The final choices of primer and probe sequences are shown highlighted in yellow and turquoise, respectively.
(PDF)

S3 File Sequences from different *H*. *capsulatum* clades used for designing primers and probes used for M antigen protocol. The final choices of primer and probe sequences are shown highlighted in yellow and turquoise, respectively.
(PDF)
