# Supplementary material for: Standardization and validation of real time PCR assays for the diagnosis of histoplasmosis using three molecular targets in an animal model
Source: PLoS One. 2017 Dec 29;12(12):e0190311. doi: 10.1371/journal.pone.0190311 (PMC5747470; doi:10.1371/journal.pone.0190311)
Supplement: S1 File — The final choices of primer and probe sequences are shown highlighted in yellow and turquoise, respectively. (PDF) [file pone.0190311.s001.pdf]

Seaview text-only output

```
               1
AJ005963rev    CAATGTCCGT TCACCAAGGT ATCCCACAGC ATCACGGAGG TATTCGGGAG ACGCGGGGAG TTGCAAGAAG
1000           .......... .......... .......... .......... .......... .......... ..........
1001           .......... .......... .......... .......... .......... .......A.. ..........
1003           .......... .......... .......... .......... .......... .......... ..........
1006           .......... .......... .......... .......... .......... .......... ..........
1008           .......... .......... .......... .......... .......... .......... ..........
2134           .......... .......... .......... .......... .......... .......... ..........
2212           .......... .......... .......... .......... .......... .......A.. ..........
2350           ...C...... .......... .......... .......... .......... .......... ..........
2353           .......... .......... .......... .......... .......... .......... ..........
2360           .......... .......... .......... .......... .......... ....A..... ..........
2365           .......... .......... .......... .......... .......... ....T..... ..........
2367           .......... .......... .......... .......... .......... .......... ..........
2404           .......... .......... .......... .......... .......... .......... ..........
2431           .......... .......... .......... .......... .......... .......... ..........
2433           .......... .......... .......... .......... .......... .......... ..........
2444           .......... .......... .......... ...C...... .......... .......... ..........
2472           .......... .......... .......... .......... .......... .......... ..........
4741           .......... .......... .......... .......... .....A.... .......... ..........
5823           .......... .......... .......... ....T..... .......... .......... ..........

             71
AJ005963rev    GAGAGAACTG TATCGGTGGC TTGGGGTCGA ATTTTCTGCA CGGAAAACTG CGGCTGCGTG AGAGGCCGGA
1000           .......... .......... .......... .......... .A........ .......... ..G.......
1001           .......... .......... .......... .......... .......... .......... ..G.......
1003           .......... .......... .......... .......... .......... .......... ..G.......
1006           .......... .......... .......... .......... .......... .......... ..G.......
1008           .......... .......... .......... .......... .......... .......... ..G.......
2134           .......... .......... .......... .......... .......... .......... ..G.......
2212           .......... .......... .......... .......... .......... .......... ..G.......
2350           .......... .......... .......... .......... .......... .......... ..G.......
2353           .......... .......... ...A...... .......... .......... .......... ..G.......
2360           .......... .......... .......... .......... .......... .......... ..G.......
2365           .......... .......... .......... .......... .......... .......... ..G.......
2367           .......... .......... ...A...... .......... .......... .......... ..G.......
2404           .......... .......... .......... .......... .......... .......... ..G.......
2431           .......... .......... .......... .......... .......... .......... ..........
2433           .......... .......... .......... .......... .......... .......... ..........
2444           .......... .......... .......... .......... .......... .......... ..G.......
2472           .......... .......... .......... .......... .......... .......... ..G.......
4741           .......... .......... .......... .......... .......... .......... ..G.......
5823           .......... .......... .......... ...C...... .......... .......... ..G.......

            141
AJ005963rev    GGCGGGTCCA CGGAACGGTT TCGGAGTTGC CGTAGTCGAT GTAAACGACG TCGGCTTTTT TCGCTTCACG
1000           .......... .......... .......... .......... .......... .......... ..........
1001           .......... .......... .......... .......... .......... .......... .A........
1003           .......... .......... .......... .......... .......... .......... ..........
1006           .......... .......... .......... .......... .......... .......... ..........
1008           .......... .......... .......... .......... .......... .......... ..........
2134           .......... .......... .......... .......... .......... .......... ..........
2212           .......... .......... .......... .......... .......... .......... .A........
2350           .......... .......... .......... .......... ......A... .......... ..........
2353           .......... .......... .......... .......... .......... .......... ..........
2360           .A........ .......... .......... .......... .......... ..A....... ..........
2365           .A........ .......... .......... .......... .......... .......... ..........
2367           .......... .......... .......... .......... .......... .......... ..........
2404           .......... .......... .......... .......... .......... .......... ..........
```

```
2431         .......... .......... .......... .......... .......... .......... ..........
2433         .......... .......... .......... .......... .......... .......... ..........
2444         .......... .......... .......... .......... .......... .......... ..........
2472         .......... .......... .......... .......... .......... .......... ..........
4741         .A........ .......... .......... .......... .......... .......... ..........
5823         .A........ .......... ..T....... .......... .......... .......... ..........

            211
AJ005963rev  GTCGTTTCTC CGTATTTTTG CGCGGTACCA CTCGTTGTCC TCCGTGAACC TGGCCGCGAC TAGATCTCCG
1000         ...A...... .......... .......... .......... .......... .......... ..........
1001         .......... .......... .......... .......... .......... .......... ..........
1003         ...A...... .......... .......... .......... .......... .......... ..........
1006         ...A...... .......... .......... .......... .......... .......... ..........
1008         ...A...... .......... .......... .......... .......... .......... ..........
2134         .......... .......... .......... .......... .......... .......... ..........
2212         .......... .......... .......... .......... .......... .......... ..........
2350         .......... .......... .......... .......... .......... .......... ..........
2353         .......... .......... .......... .......... .......... .......... ..........
2360         .......... .......... .......... .......... .......... .......... ..........
2365         .......... .......... .......... .......... .......... .......... ..........
2367         .......... .......... .......... .......... .......... .......... ..........
2404         ...A...... .......... .......... .......... .......... .......... ..........
2431         .......... .......... .......... .......... .......... .......... ..........
2433         .......... .......... .......... .......... .......... .......... ..........
2444         .......... .......... .......... .......... .......... .......... ..........
2472         ...A...... .......... .......... .......... .......... .......... ..........
4741         ........G. .......... .......... .......... .......... .......... ..........
5823         .......... .......... .......... .......... .......... .......... C.........

            281
AJ005963rev  GCCTTGGGTG GGCCGCTCAG AG
1000         .......... .......... ..
1001         .......... .......... ..
1003         .......... .......... ..
1006         .......... .......... ..
1008         .......... .......... ..
2134         .......... .......... ..
2212         .......... .......... ..
2350         .......... .......... ..
2353         .......... .......... ..
2360         .......... .......... ..
2365         .......... .......... ..
2367         .......... .......... ..
2404         .......... .......... ..
2431         .......... .......... ..
2433         .......... .......... ..
2444         .......... .......... ..
2472         .......... .......... ..
4741         ........G. CC........ ..
5823         .......... .......... ..
```
