# Supplementary material for: Standardization and validation of real time PCR assays for the diagnosis of histoplasmosis using three molecular targets in an animal model
Source: PLoS One. 2017 Dec 29;12(12):e0190311. doi: 10.1371/journal.pone.0190311 (PMC5747470; doi:10.1371/journal.pone.0190311)
Supplement: S2 File — The final choices of primer and probe sequences are shown highlighted in yellow and turquoise, respectively. (PDF) [file pone.0190311.s002.pdf]

Seaview text-only output

```
             1
U20346rev    GAGGAGCACC GAGATAAAGG TGTCGACGTT G--T-ATTGG GCCCTGTGGT TGGACCGTTG GGCAGGTCGC
1001         .......... .......... .......... ....G..... .T........ .......... ..........
2763         .......... .......... .......... .......... .T........ .......... ..........
2212         .......... .......... .......... .A..T..... .T........ .......... ..........
5822         .......... .......... .......... .A..T..... .T........ .......... ..........
2357         .......... .......... .......... ....T..... .T........ .......... ..........
2349         .......... .......... .......... .......... .......... .......... ..........
2368         .......... .......... .......... .......... .......... .......... ..........
2475         .......... .......... .......... .......... .......... .......... ..........
1006         .......... .......... .......... ....G..... .......... .......... ..........
2474         .......... .......... .......... .......... .......... .......... ..........
2358         .......... .......... .......... .......... .......... .......... ..........
2433         .......... .......... .......... ........A. .......... .......... ..........
2359         .......... .......... .......... .A..T..... .......... .......... ..........
2365         .......... .......... .......... .A..T..... .......... .......... ..........
2363         .......... .......... .......... .A..T..... .......... .......... ..........
2434         .......... .......... .......... ....T..... .......... .......... ..........
2404         .......... .......... .......... ....T..... .......... .......... ..........
2431         .......... .......... .......... ....T..... .......... .......... ..........
2472         .......... .......... .......... ...-T..... .......... .......... ..........
1008         .......... .......... .......... ....T..... .......... .......... ..........
2436         .......... .......... .......... .......... .......... .......... ..........
1003         .......... .......... .......... .......... .......... .......... ..........
2444         .......... .......... ........A. TG.-T..... .T........ .......... ..........
4741         .......... .......... ........-. TG.-T..... .T........ .......... ..........
5823         .......... .......... .......... .AA.T..... .T........ .......... ..........
2134         .......... .......... .........- -T.GT..... .T........ .......... ..........
2350         .......... .......... .........G AT.GT..... .T........ .......... ..........
2367         .......... .......... .........- -T.GT..... .T........ .......... ..........
2355         .......... .......... .......... .T.-T..... .T........ .......... ..........
2352         .......... .......... .......... -..GT..... .T........ .......... ..........
2353         .......... .......... .........A TG.GT..... .T........ .......... ..........

            71
U20346rev    CAGATGGAGG GCGGAATTGG GAAGGTTTCA GCCCTGATCC TGTCAATTCC GGGCTCCTTG TTGCGGAGAC
1001         .......... .......... .......... .......... .......... .......... ....A.....
2763         .......... .......... .......... .......... .......... .......... ....A.....
2212         .......... .......... .......... .......... .......... .......... ....A.....
5822         .......... .......... .......... .......... .......... .......... ....A.....
2357         .......... .......... .......... .......... .......... .......... ....A.....
2349         .......... .......... .......... .......... .......... .......... ..........
2368         .......... .......... .......... .......... .......... .......... ..........
2475         .......... .......... .......... .......... .......... .......... ..........
1006         .......... .......... .......... .......... .......... .......... ..........
2474         .......... .......... .......... .......... .......... .......... ..........
2358         .......... .......... .......... .......... .......... .......... ..........
2433         .......... .......... .......... .......... .......... .......... ..........
2359         .......... .......... .......... .......... .......... .......... ..........
2365         .......... .......... .......... .......... .......... .......... ..........
2363         .......... .......... .......... .......... .......... .......... ..........
2434         .......... .......... .......... .......... .......... .......... ..........
2404         .......... .......... .......... .......... .......... .......... ..........
2431         .......... .......... .......... .......... .......... .......... ..........
```

```
2472        .......... .......... .......... .......... .......... .......... ..........
1008        .......... .......... .......... .......... .......... .......... ..........
2436        .......... .......... .......... .......... .......... .......... ..........
1003        .......... .......... .......... .......... .......... .......... ..........
2444        .......... .......... .......... .......... .......... .......... ..........
4741        .......... .......... .......... .......... .......... .......... ..........
5823        .......... ...A...... .......... .......... .........T .......... ..........
2134        .......... .......... .......... .......... .......... .......... ..........
2350        .......... .......... .......... .......... .......... .......... ..........
2367        .......... .......... .......... .......... .......... .......... ..........
2355        .......... .......... .......... .......... .......... .......... ..........
2352        .......... .......... .......... .......... .......... .......... ..........
2353        .......... .......... .......... .......... .......... ...A...... ....A.....

         141
U20346rev   CATCAAGGGA ATTCAAAGTG CCGGCGTCAT TGCTTGTGTC AAGCATTTCA TAGGCAACGA GCAAGAACGA
1001        .......... .......... ..A....... .......... .......... .......... ......G...
2763        .......... .......... ..A....... .......... .......... .......... ......G...
2212        .......... .......... ..A....... .......... .......... .......... ......G...
5822        .......... .......... ..A....... .......... .......... .......... ......G...
2357        .......... .......... ..A....... .......... .......... .......... ......G...
2349        .......... .......... .......... .......... .......... .......... ......G...
2368        .......... .......... .......... .......... .......... .......... ......G...
2475        .......... .......... .......... .......... .......... .......... ......G...
1006        .......... .......... .......... .......... .......... .......... ......G...
2474        .......... .......... .......... .......... .......... .......... ......G...
2358        .......... .......... .......... .......... .......... .......... ......G...
2433        .......... .......... ........G. .......... .......... .......... ......G...
2359        .......... .......... .......... .......... .......... .......... ......G...
2365        .......... .......... .......... .......... .......... .......... ......G...
2363        .......... .......... .......... .......... .......... .......... ......G...
2434        .......... .......... .......... .......... .......... .......... ......G...
2404        .......... .......... .......... .......... .......... .......... ......G...
2431        .......... .......... .......... .......... .......... .......... ......G...
2472        .......... .......... .......... .......... .......... .......... ......G...
1008        .......... .......... .......... .......... .......... .......... ..........
2436        .......... .......... .......... .......... .......... .......... ..........
1003        .......... .......... .......... .......... .......... .......... ......G...
2444        .......... .......... .......... .......... .......... .......... ......G...
4741        .......... .......... .......... .......... .......... .......... ......G...
5823        .......... .......... .......A.. .......... .......... .......... ......G...
2134        .......... .......... .......... .......... .......... .......... ......G...
2350        .......... .......... .......... .......... .......... .......... ......G...
2367        .......... .......... .......... .......... .......... .......... ......G...
2355        .......... .......... .......... .......... .......... .......... ......G...
2352        .......... .......... .......... .......... .......... .......... ......G...
2353        .......... .......... .......... .......... .......... .......... ......G...

         211
U20346rev   TTCCGCCAAG GCCCTGAGGC TCAAGGGTAT GGTTTCGATA TCTCAGAGAG T--TCAAGTT CCAATATTGA
1001        ........G. .......... .......... .......... .......... .......... ..........
2763        ........G. .......... .......... .......... .......... .......... ..........
2212        ........G. .......... .......... .......... .......... .......... ..........
5822        ........G. .......... .......... .......... .......... .CA....... ..........
2357        ........G. .......... .......... .......... .......... .......... ..........
```

```
2349         .......... .......... .......... .......... .......... .AT....... ..........
2368         .......... .......... .......... .......... .......... .......... ..........
2475         .......... .......... .......... .......... .......... .......... ..........
1006         .......... .......... .......... .......... .......... .......... ..........
2474         .......... .......... .......... .......... .......... .......... ..........
2358         .......... .......... .......... .......... .......... .......... ..........
2433         .......... .......... .......... .......... .......... .......... ..........
2359         .......... .......... .......... .......... .......... .......... ..........
2365         .......... .......... .......... .......... .......... .......... ..........
2363         .......... .......... .......... .......... .......... .......... ..........
2434         .......... .......... .......... .......... .......... .......... ..........
2404         .......... .......... .......... .......... .......... .......... ..........
2431         .......... .......... .......... .......... .......... .......... ..........
2472         .......... .......... .......... .......... .......... .......... ..........
1008         .......... .......... .......... .......... .......... .......... ..........
2436         .......... .......... .......... .......... .......... .......... ..........
1003         .......... .......... .......... .......... .......... .CA....... ..........
2444         ........G. .......... .......... .......... .......... .......... ..........
4741         ........G. .......... .......... .......... .......... .......... ..........
5823         ........G. .......... .......... .......... .......... C......... ..........
2134         .....T..G. ....A..... ...G...... .......... .......... ..C....... ..........
2350         .....T..G. ....A..... ...G...... .......... .......... ..A....... ..........
2367         .....T..G. ....A..... ...G...... .......... .......... ..A....... ..........
2355         .....T..G. ....A..... ...G...... .......... .......... ..A....... ..........
2352         .....T..G. .......... .......... .......... .......... .......... ..........
2353         .....T..G. .......... .......... .......... .......... .CA....... ..........

          281
U20346rev  CGACGTAACC ATGCA
1001         .......... .....
2763         .......... .....
2212         .......... .....
5822         .......... .....
2357         .......... .....
2349         .......... .....
2368         .......... .....
2475         .......... .....
1006         .......... .....
2474         .......... .....
2358         .......... .....
2433         .......... .....
2359         .......... .....
2365         .......... .....
2363         .......... .....
2434         .......... .....
2404         .......... .....
2431         .......... .....
2472         .......... .....
1008         .......... .....
2436         .......... .....
1003         .......... .....
2444         .......... .....
4741         .......... .....
5823         .......... .....
2134         .......... .....
```

2350	....	.....
2367	....	.....
2355	....	.....
2352	....	.....
2353	....	.....
