# Supplementary material for: Standardization and validation of real time PCR assays for the diagnosis of histoplasmosis using three molecular targets in an animal model
Source: PLoS One. 2017 Dec 29;12(12):e0190311. doi: 10.1371/journal.pone.0190311 (PMC5747470; doi:10.1371/journal.pone.0190311)
Supplement: S3 File — The final choices of primer and probe sequences are shown highlighted in yellow and turquoise, respectively. (PDF) [file pone.0190311.s003.pdf]

Seaview text-only output

```
         1
1001     CCCATCGCTT GATCCCCAAC GCGGCGTGAA CATGACCTAT TCCGGCGCCG ACGGCTCGAT CTTCGATGCC
4741     .......... .......... .......... .......... .......... .......... T........T
1006     .T........ .......... .......... .......... .......... .......... ..........
2472     .T........ .......... .......... .......... .......... .......... ..........
1000     .T........ .......... .......... .......... .......... .......... ..........
2404     .T........ .......... .......... .......... .......... .......... ..........
2436     .T........ .......... .......... .......... .......... .......... ..........
1008     .T........ .......... .......... .......... .......... .......... ..........
1003     .T........ .......... .......... .......... .......... .......... ..........
2434     .T........ .......... .......... .......... .......... .......... ..........
2474     .T........ .......... .......... .......... .......... .......... ..........
5822     .T........ .......... .......... .......... .......... .......... ..........
2475     .......... .......... .......... .......... .......... .......... ..........
2212     .......... .......... .......... .......... .......... .......... ..........
2360     T......... .......... .......... .......... .......... .......... ..........
5823     .......... .......... .......... .......... .......... .......... ..........
2350     .......... .......... .......... .......... .......... .......... ..........
2352     .......... .......... .......... .......... .......... .......... ..........
2433     T......... .......... .......... .......... .......... .......... ..........
2444     .......... .......... .......... .......... .......... .......... ..........
2358     .......... .......... .......... .......... .......... .......... ..........
2365     .......... .......... .......... .......... .......... .......... ..........
2357     .......... .......... .......... .......... .......... .......... ..........
2367     .......... .......... .......... .......... .......... .......... ..........
2363     .......... .......... .......... .......... .......... .......... ..........
2349     .......... .......... .......... .......... .......... .......... ..........
2353     .......... .......... .......... .......... .......... .......... ..........

        71
1001     GTGATCGTCG TCGGCGGCCT GCTCACGAGC GCCTCAACGC AATACCCAAC GGGTCGCCCG CTCAGGATTA
4741     .......... .......... .......... .......... .......... .......... .....A....
1006     .......... .......... .......... .......... .........G A......... ..........
2472     .......... .......... .......... .......... .........G A......... ..........
1000     .......... .......... .......... .......... .........G A......... ..........
2404     .......... .......... .......... .......... .........G A......... ..........
2436     .......... .......... .......... .......... .........G A......... ..........
1008     .......... .......... .......... .......... .........G A......... ..........
1003     .......... .......... .......... .......... .........G A......... ..........
2434     .......... .......... .......... .......... .........G A......... ..........
2474     .......... .......... .......... .......... .........G A......... ..........
5822     .......... .......... .......... .......... .........G A......... ..........
2475     .......... .......... .......... .......... .........G A......... ..........
2212     .......... .......... .......... .......... .......... .......... ..........
2360     .......... ........T. ....C..... A......... .......... .......... ..........
5823     .......... .......... .......... .......... .......... .......... ..........
2350     .......... .......... .......... .......... .......... .......... ..........
2352     .......... .......... .......... .......... .......... .......... ..........
2433     .......... ........T. ....C..... A......... .......... .......... ..........
2444     .......... .T........ .......... T......... .......... .......... ..........
2358     .......... .......... ........A. .......... .......... .......... ..........
2365     .......... ..A....... ........A. .......... .......... .......... ..........
2357     .......... .......... ........A. .......... .......... .......... ..........
2367     .......... ..A....... ........A. .......... .......... .......... ..........
2363     .......... ..A....... ........A. .......... .......... .......... ..........
2349     .......... ..A....... ........A. .......... .......... .......... ..........
2353     .......... .......... .......... .......... .......... .......... ..........
```

```
       141
1001     TTACGGATGC ATACGCGTAT GGAAAGCCCG TTGGCGCCGT CG
4741     .......... .......... .......... .......... ..
1006     .......... .......... .......... .......... ..
2472     .......... .......... .......... .......... ..
1000     .......... .......... .......... .......... ..
2404     .......... .......... .......... .......... ..
2436     .......... .......... .......... .......... ..
1008     .......... .......... .......... .......... ..
1003     .......... .......... .......... .......... ..
2434     .......... .......... .......... .......... ..
2474     .......... .......... .......... .......... ..
5822     .......... .......... .......... .......... ..
2475     .......... .......... .......... .......... ..
2212     .......... .......... .......... .......... ..
2360     .......... ....A..... .......... .......... ..
5823     .......... .......... .......... .......... ..
2350     .......... ....AT.... .......... .......... ..
2352     .......... ....AT.... .......... .......... ..
2433     .......... ....A..... .......... .......... ..
2444     ...T...... .......... .......... .C........ ..
2358     .......... .......... .......... .......... ..
2365     .......... .......... .......... .......... T.
2357     .......... .......... .......... .......... T.
2367     .......... .......... .......... .......... T.
2363     .......... .......... .......... .......... T.
2349     .......... .......... .......... .......... T.
2353     .......... .......... .......... .......... ..
```
